# LncRNA *ZNF674-AS1* regulates granulosa cell glycolysis and proliferation by interacting with ALDOA

**DOI:** 10.1038/s41420-021-00493-1

**Published:** 2021-05-16

**Authors:** Duan Li, Xiaoyan Wang, Guangyu Li, Yujie Dang, Shidou Zhao, Yingying Qin

**Affiliations:** 1 Center for Reproductive Medicine, Cheeloo College of Medicine, Shandong University, 250012 Jinan, Shandong China; 2 Key laboratory of Reproductive Endocrinology of Ministry of Education, Shandong University, 250012 Jinan, Shandong China; 3 Shandong Key Laboratory of Reproductive Medicine, 250012 Jinan, Shandong China; 4 Shandong Provincial Clinical Research Center for Reproductive Health, 250012 Jinan, Shandong China; 5 National Research Center for Assisted Reproductive Technology and Reproductive Genetics, Shandong University, 250012 Jinan, Shandong China

**Keywords:** Post-translational modifications, Endocrine reproductive disorders

## Abstract

Granulosa cell (GC) is a critical somatic component of ovarian follicles to support oocyte development, while the regulatory role of long noncoding RNA (lncRNA) in GCs is largely unknown. Here, we identified a down-regulated lncRNA *ZNF674-AS1* in GCs from patients with biochemical premature ovarian insufficiency (bPOI), and its expression correlates with serum levels of clinical ovarian reserve indicators. Functional experiments showed that *ZNF674-AS1* is induced by energy stress, and regulates the proliferation and glycolysis of GCs, which possibly leads to follicular dysfunction. Mechanistically, low-expressed *ZNF674-AS1* reduced the enzymatic activity of aldolase A (ALDOA), concomitant with promoting the association between ALDOA and v-ATPase to activate the lysosome localized AMP-activated protein kinase (AMPK). These findings identified a new lncRNA–ALDOA complex through which *ZNF674-AS1* exerts its functions, expanding the understanding of epigenetic regulation of GCs function and POI pathogenesis.

## Introduction

In mammals, the ovarian follicle is the basic female reproductive unit which consists of oocytes and two types of somatic cells, granulosa cells (GCs) and theca cells^1^. GCs are supposed to provide physical and hormonal support to oocytes so that folliculogenesis can be successfully executed to fulfill female fertility^2,3^. Notably, GCs apoptosis initiates follicular atresia^4^. Follicular growth originates from primordial follicle formation to the final ovulation. Any elements that disrupt this process will diminish the ovarian reserve pool and/or accelerate ovarian follicle depletion, therefore resulting in premature ovarian insufficiency (POI)^5^, which is defined as partial or complete loss of ovarian function before the age of 40 years^6^. POI is a heterogeneous disorder affecting ~1% of women of reproductive age^6^. Clinically, three stages of POI have been described, including occult, biochemical, and eventually overt^5,7,8^. Patients with biochemical POI (bPOI) show raised concentrations of follicle-stimulating hormone (FSH), in whom the ovarian reserve has not yet been fully exhausted. Recognized causes of POI include genetic, autoimmune, and iatrogenic factors^9^, however, the majority remains unclear.

Long noncoding RNAs (lncRNAs) are transcripts longer than 200 nucleotides without protein-coding function^10^, which performs diverse regulatory roles in many biological processes, such as gene regulation, mRNA splicing, and protein stabilization^11,12^. Aberrant expression of lncRNAs has been observed in many pathological conditions, regulating tumor metastasis^13^, cell viability^14^, metabolism alteration^15^, and innate immunity^16^. However, the regulatory roles of lncRNAs in the GCs function are not completely characterized. Therefore, elucidating the role of lncRNAs underlying GCs function will expand the understanding of the molecular mechanisms of follicular development and benefit the diagnosis and treatment of POI.

In the present study, we identified a down-regulated lncRNA *ZNF674-AS1* in GCs from patients with POI and demonstrated its essential regulatory role in the glycolysis and proliferation of GCs. *ZNF674-AS1* functions through interactions with aldolase A (ALDOA), a key enzyme that modulates the glycolysis process^17^ and controls the activation of AMP-activated protein kinase (AMPK) at lysosome^18–20^. Moreover, our findings suggested that expression of *ZNF674-AS1* in GCs correlates with serum levels of clinical ovary function indicators and that down-regulated *ZNF674-AS1* might contribute to the development of POI.

## Results

### LncRNA *ZNF674-AS1* is down-regulated in GCs from patients with bPOI and correlates with poor ovarian reserve

**Fig. 1:**
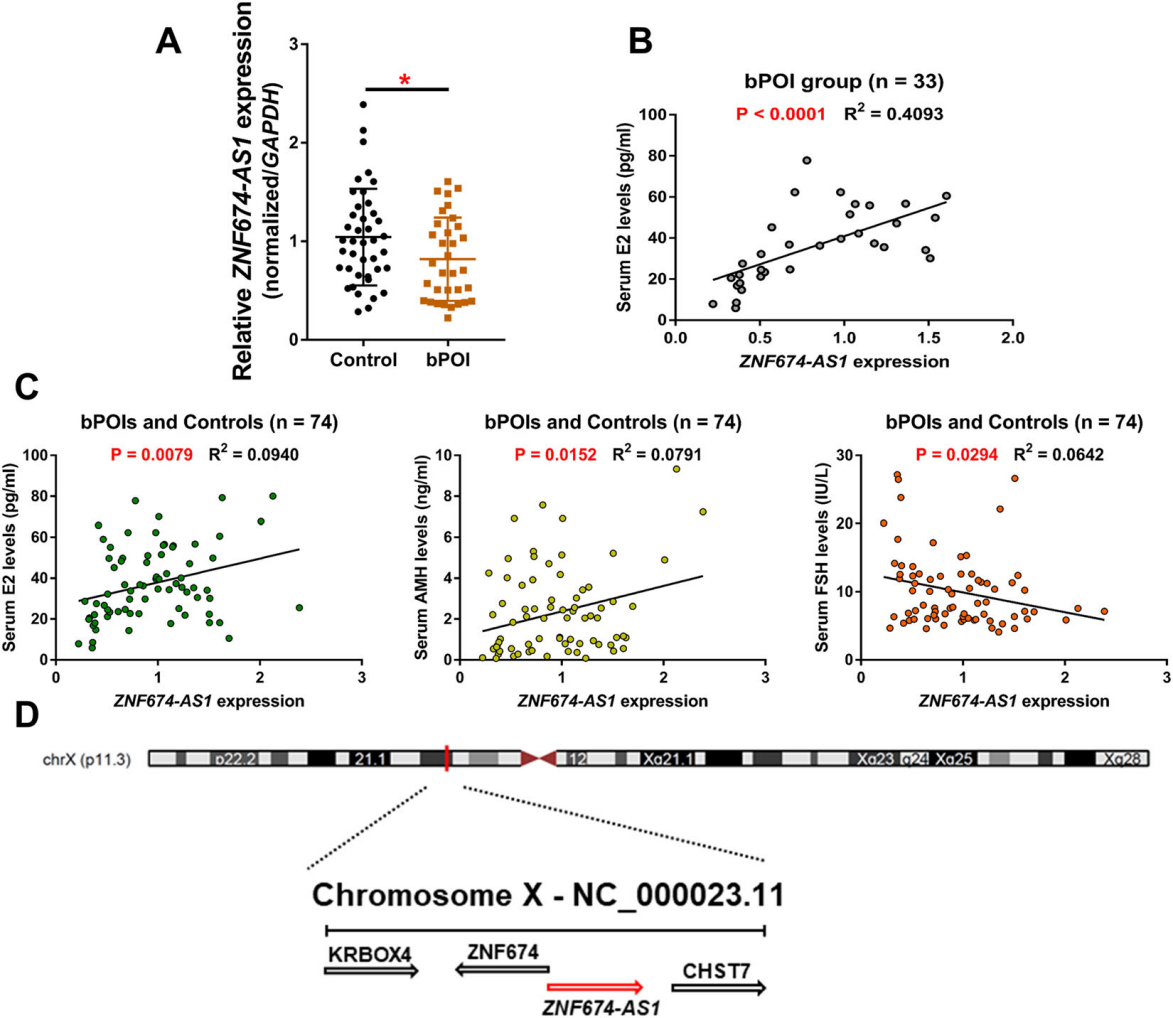
LncRNA *ZNF674-AS1* is down-regulated in GCs from patients with bPOI and correlates with poor ovary reserve. **A** The expression level of *ZNF674-AS1* was validated by qRT-PCR in GCs from patients with bPOI (*n* = 33) and controls (*n* = 41). Ct values were normalized to GAPDH. *p < 0.05, by two-tailed Student’s *t* test. **B** The correlation between the expression level of *ZNF674-AS1* in GCs and the serum concentration of estradiol from patients with bPOI (*n* = 33) was analyzed by Pearson correlation analysis. **C** The correlation between the expression level of *ZNF674-AS1* in GCs and the serum concentration of estradiol, AMH, FSH from all participants (*n* = 74) was analyzed by Pearson correlation analysis. **D** Schematic representation of localization of *ZNF674-AS1* in chromosome X.

To uncover lncRNAs that are differentially expressed in GCs from patients with bPOI, we collected the GCs from eight patients with bPOI and nine age/BMI-matched healthy women for RNA-seq analysis (GEO: GSE158526). Results showed that 78 lncRNAs were differentially expressed in GCs from patients with bPOI (Supplementary Table 1). Subsequently, six lncRNAs (*AC112721.1*, *ZNF674-AS1*, *RP4-545C24.1*, *GS1-358P8.4*, *SNAI3-AS1*, and *RP11-3D4.3*) with highly conserved properties based on UCSC^21^ resources were singled out for qRT-PCR validation, and the down-regulated lncRNA *ZNF674-AS1*, which was validated in GC samples from 33 patients with bPOI and 41 age-matched controls (Fig. 1A), stood out by showing a significant correlation with serum levels of clinical ovary function indicators. In detail, the expression of *ZNF674-AS1* showed a positive correlation with serum levels of basic estradiol in 33 patients with bPOI (Fig. 1B and Supplementary Fig. 1A). Furthermore, correlation analysis in all participants (bPOIs and Controls, *n* = 74) showed that the expression of *ZNF674-AS1* was positively correlated with serum levels of basic estradiol and AMH, and negatively correlated with serum levels of basic FSH (Fig. 1C), suggesting the regulatory role of *ZNF674-AS1* in ovary functions. Therefore, lncRNA *ZNF674-AS1* was selected for further exploration. LncRNA *ZNF674-AS1* is located at chromosome X with limited known functions (Fig. 1D). Coding Potential Assessment Tool showed the noncoding feature of lncRNA *ZNF674-AS1* (Supplementary Fig. 1B). Collectively, lncRNA *ZNF674-AS1* is down-regulated in patients with bPOI and *ZNF674-AS1* expression is a promising indicator for ovarian reserve.

### Silencing of lncRNA *ZNF674-AS1* significantly inhibited the proliferation of GCs

**Fig. 2:**
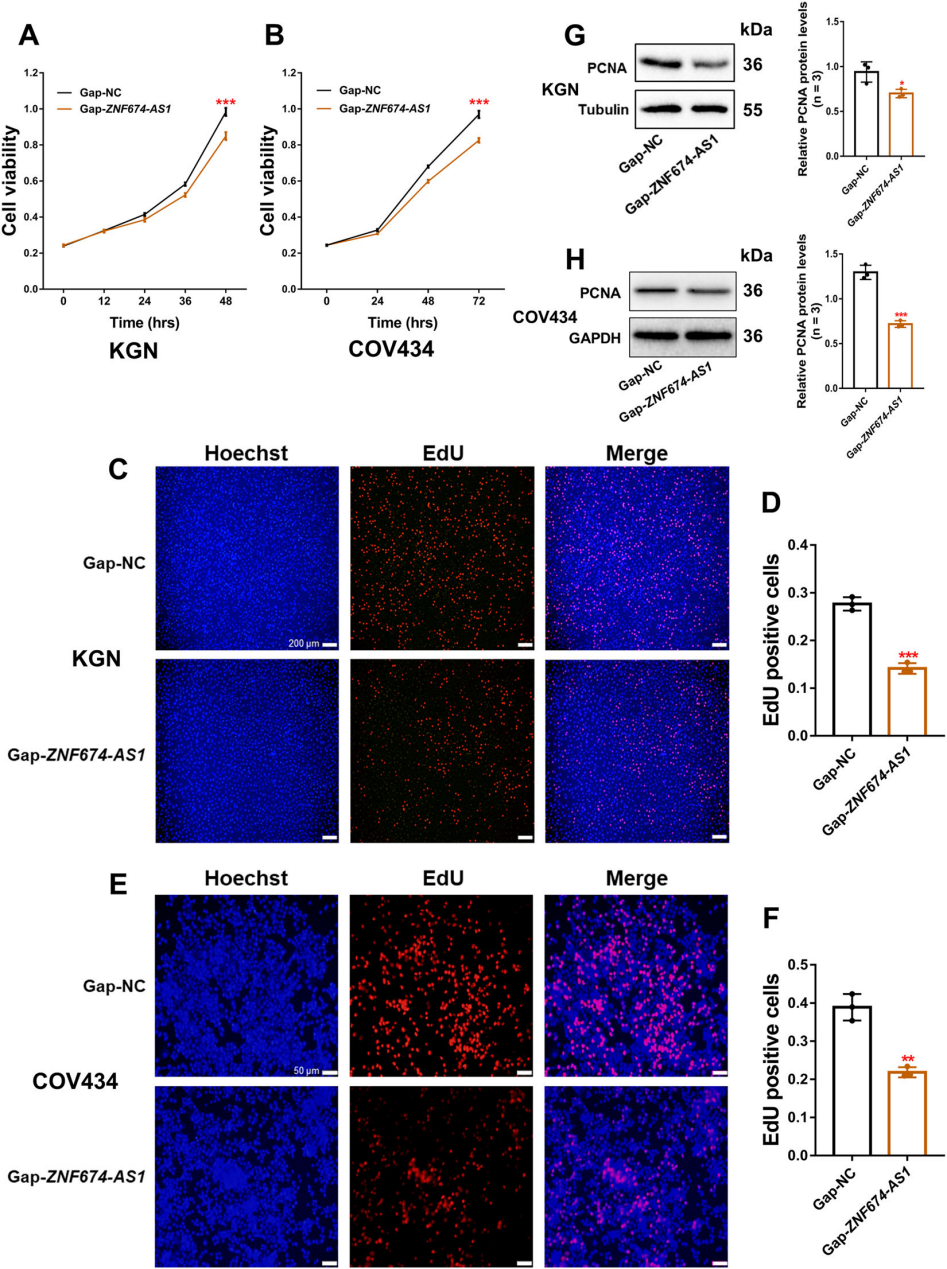
Silencing of lncRNA *ZNF674-AS1* significantly inhibited the proliferation of GCs. **A**, **B** The viability of KGN and COV434 cells after *ZNF674-AS1* silencing was assessed by CCK8 assay. Results are expressed as the mean ± SD (*n* = 3). ***p < 0.001 by two-tailed Student’s *t* test. **C**, **D** The proliferation of KGN cells after *ZNF674-AS1* silencing was evaluated by EdU staining assay. Results are expressed as the mean ± SD (*n* = 3). ***p < 0.001 by two-tailed Student’s *t* test. Scale bar: 200 μm. **E**, **F** The proliferation of COV434 cells after *ZNF674-AS1* silencing was evaluated by EdU staining assay. Results are expressed as the mean ± SD (*n* = 3). **p < 0.01 by two-tailed Student’s *t* test. Scale bar: 50 μm. **G**, **H** PCNA protein levels in KGN and COV434 cells were analyzed by western blot after *ZNF674-AS1* silencing. For gray value quantification of PCNA, data were normalized to the internal reference. Shown are mean ± SD. *p < 0.05, ***p < 0.001 by two-tailed Student’s *t* test.

To explore the roles of lncRNA *ZNF674-AS1* on GC function, we silenced *ZNF674-AS1* in KGN and COV434 cells via antisense LNA GapmeRs (Supplementary Fig. 1C). Decreased cell viability of KGN and COV434 cells was observed after *ZNF674-AS1* knockdown (Fig. 2A, B). EdU assays showed that *ZNF674-AS1* silence significantly inhibited the proliferation of KGN and COV434 cells (Fig. 2C–F). Furthermore, protein levels of proliferating cell nuclear antigen were decreased in *ZNF674-AS1* silenced KGN and COV434 cells (Fig. 2G, H). Taken together, these results indicated that silencing lncRNA *ZNF674-AS1* inhibited GC proliferation.

We also detected the impact of *ZNF674-AS1* knockdown on steroid hormone synthesis of KGN cells. However, results showed that levels of estradiol, as well as FSHR, CYP19A1 proteins, remain unchanged in *ZNF674-AS1* silenced KGN cells (Supplementary Fig. 1D, E).

### LncRNA *ZNF674-AS1* interacts with ALDOA protein

**Fig. 3:**
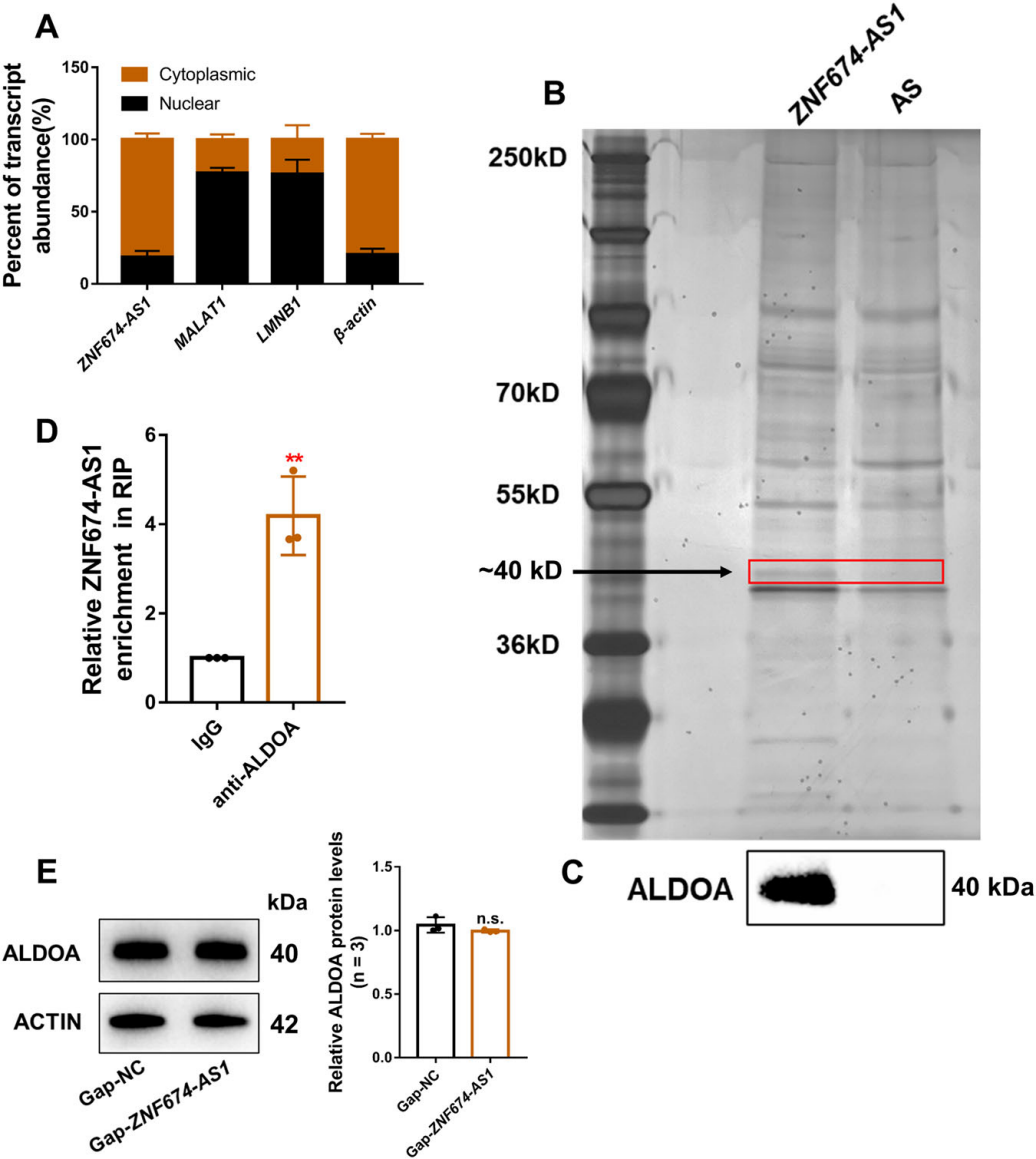
LncRNA *ZNF674-AS1* interacts with ALDOA protein. **A** The relative expression levels of *ZNF674-AS1* in the cytoplasmic and nuclear fractions of KGN cells were detected by qRT-PCR. *MALAT1* was the nuclear lncRNA control, Lamin B1 was the nuclear control, and *GAPDH* was the cytoplasmic control. **B** A specific band of ∼40 kDa (red box) were pulled down by *ZNF674-AS1* and subsequently analyzed by mass spectrometry. **C** The specific association between *ZNF674-AS1* and ALDOA was validated by western blot in the samples obtained from RNA pull-down. The antisense strand of *ZNF674-AS1* was used as the negative control. **D** RIP assay was utilized to confirm the interaction between *ZNF674-AS1* and ALDOA in KGN cells. Results are expressed as the mean ± SD (*n* = 3). **p < 0.01 by two-tailed Student’s *t* test. **E** ALDOA protein levels in KGN cells were analyzed by western blot after *ZNF674-AS1* silencing. For gray value quantification of ALDOA, data were normalized to the internal reference. Shown are mean ± SD. n.s. no significance by two-tailed Student’s *t* test.

Subcellular fraction assay revealed that *ZNF674-AS1* was predominantly distributed in the cytoplasm (Fig. 3A). To uncover the molecular mechanism of *ZNF674-AS1* function, an RNA pulldown assay was performed to identify proteins that directly binds with *ZNF674-AS1*. After separated on SDS–PAGE and visualized by silver staining, a ~40 kDa band was specifically present in *ZNF674-AS1* pulldown samples (Fig. 3B), which was cut out and subjected to mass spectrometry. Subsequently, ALDOA was identified as the protein binding with *ZNF674-AS1* after analysis of mass spectrometry followed by western blot validation (Fig. 3C). To corroborate these findings, we used antibodies against ALDOA to immunoprecipitate endogenous RNAs from the total lysates of KGN cells. We observed a significant enrichment of *ZNF674-AS1* in the anti-ALDOA immunoprecipitates compared with the IgG control (Fig. 3D). Therefore, we explored whether *ZNF674-AS1* plays a role in the expression or subcellular localization of ALDOA protein. Western blot and immunofluorescence showed that silencing of *ZNF674-AS1* had no affect the protein level and the subcellular localization of ALDOA (Fig. 3E and Supplementary Fig. 1F). Taken together, these results indicated that *ZNF674-AS1* and ALDOA directly bind with each other.

### LncRNA *ZNF674-AS1* is involved in the glycolysis and glucose metabolism of GCs

**Fig. 4:**
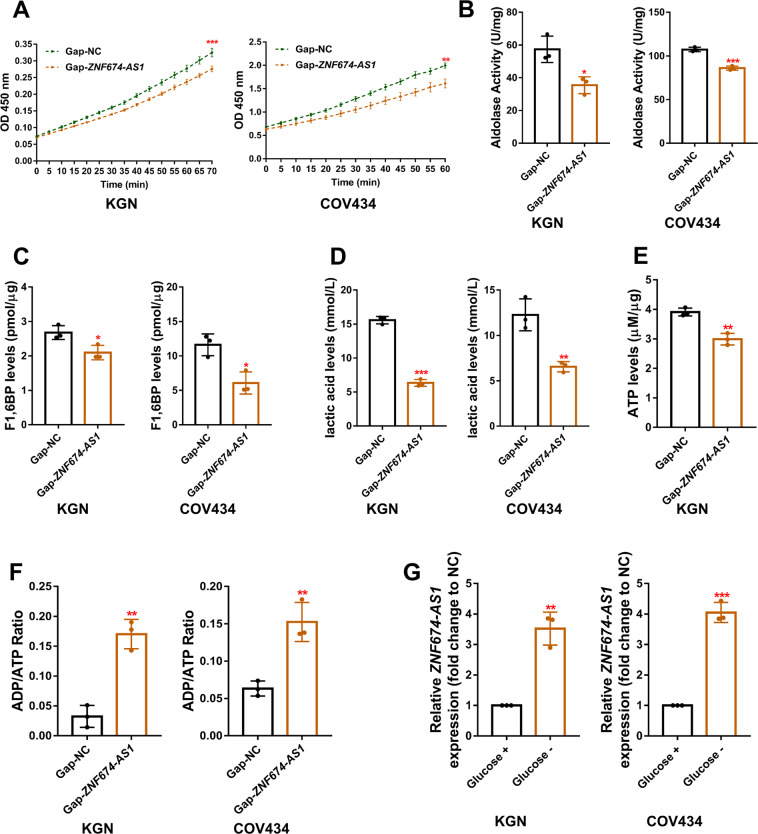
LncRNA *ZNF674-AS1* is involved in the glycolysis and glucose metabolism of GCs. **A**, **B** The aldolase activities of KGN and COV434 cells after ZNF674-AS knockdown were examined by an Aldolase Activity Colorimetric Assay Kit and compared with control cells. Results are normalized to the total protein and expressed as the mean ± SD (*n* = 3), *p < 0.05, **p < 0.01, ***p < 0.001 by two-sided Student’s *t* test. **C** Levels of F1,6BP in KGN and COV434 cells after *ZNF674-AS1* silencing were measured and normalized to the total protein. Results are expressed as the mean ± SD (*n* = 3), *p < 0.05, by the two-sided Student’s *t* test. **D** Lactic acid production in *ZNF674-AS1* silenced and negative control KGN and COV434 cells was measured via Lactate Colorimetric Assay Kit. Results are expressed as the mean ± SD (*n* = 3), **p < 0.01, ***p < 0.001 by two-sided Student’s *t* test. **E** Levels of ATP in *ZNF674-AS1* silenced and negative control KGN cells were measured and normalized to the total protein. Results are expressed as the mean ± SD (*n* = 3), **p < 0.01 by two-tailed Student’s *t* test. **F** The ratio of ADP/ATP in *ZNF674-AS1* silenced and negative control KGN and COV434 cells were detected. Results are expressed as the mean ± SD (*n* = 3), **p < 0.01 by two-tailed Student’s *t* test. **G** The expression levels of *ZNF674-AS1* were analyzed by qRT-PCR in glucose-containing and glucose-free medium cultured KGN and COV434 cells. Results were expressed as the mean ± SD (*n* = 3). **p < 0.01, ***p < 0.001 by two-tailed Student’s *t* test.

As reported, ALDOA plays a vital role in the glycolysis process, which converts fructose-1, 6-bisphosphate (F1,6BP) into glyceraldehyde-3-phosphate and dihydroxyacetone phosphate^17^. Therefore, we investigated whether *ZNF674-AS1* regulates the enzymatic activity of ALDOA. As shown in Fig. 4A, B, the aldolase activity was obviously reduced in *ZNF674-AS1* silenced KGN and COV434 cells. Moreover, we detected the concentrations of F1,6BP and lactic acid, middle and terminal products of glycolysis, to evaluate the influence of *ZNF674-AS1* silence on the glycolysis of GCs. Results showed that the concentrations of F1,6BP and lactic acid were significantly decreased after *ZNF674-AS1* knockdown (Fig. 4C, D), indicating the regulation of *ZNF674-AS1* on glycolysis. Moreover, the ATP levels was reduced (Fig. 4E) and the ratio of ADP/ATP was significantly elevated in *ZNF674-AS1* silenced KGN and COV434 cells (Fig. 4F), suggesting the abnormal process of glucose metabolism upon *ZNF674-AS1* knockdown. Based on the results above, we hypothesized that lncRNA *ZNF674-AS1* is necessary for the glycolysis and the glucose metabolism of GCs. To validate our hypothesis, we explored whether the energy status influences the expression of *ZNF674-AS1*. We detected the expression of *ZNF674-AS1* in glucose-containing and glucose-free medium cultured cells. As shown in Fig. 4G, *ZNF674-AS1* was significantly up-regulated in glucose-free medium cultured KGN and COV434 cells. Taken together, these data indicated that energy stress-induced *ZNF674-AS1* is necessary for the glycolysis process and the glucose metabolism of GCs.

### The silence of *ZNF674-AS1* promotes the activation of AMPK

**Fig. 5:**
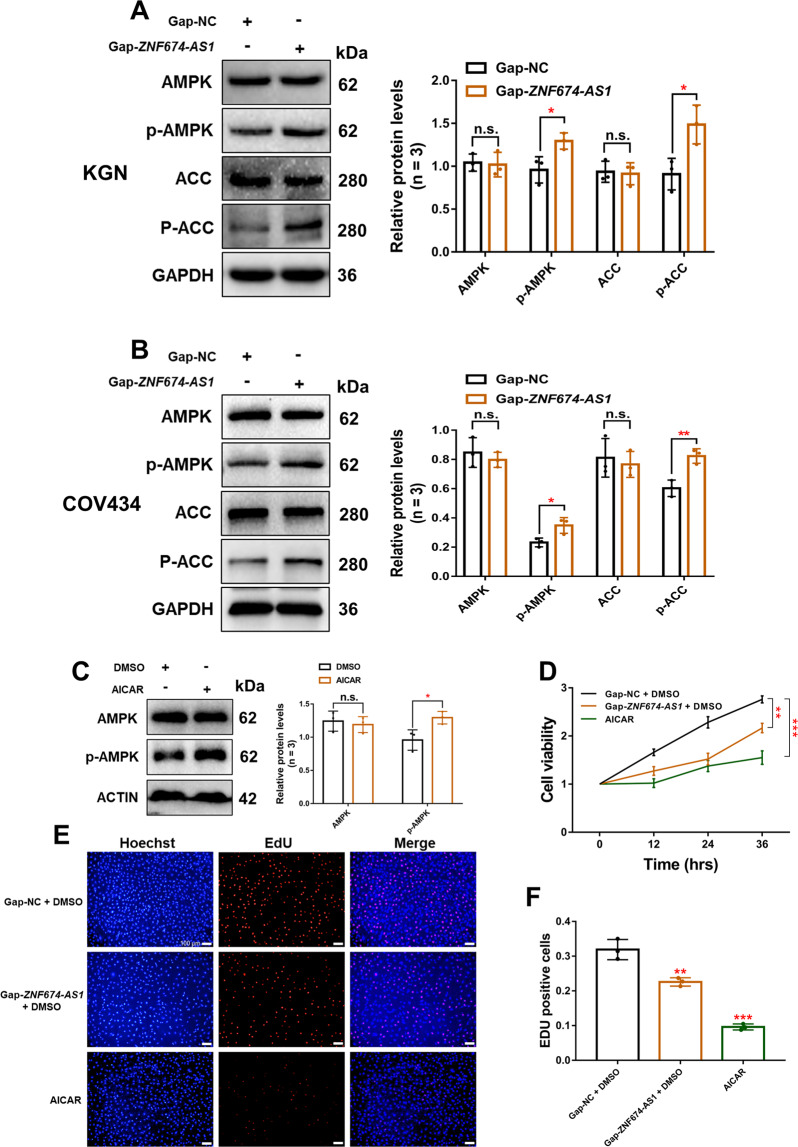
The silence of *ZNF674-AS1* promotes the activation of AMPK. **A**, **B** Western blot showing the levels of phosphorylated proteins of the AMPK signaling pathway in *ZNF674-AS1* silenced and negative control KGN and COV434 cells. For gray value quantification of proteins, data were normalized to the internal reference. Shown are mean ± SD. n.s. no significance, *p < 0.05, **p < 0.01 by two-tailed Student’s *t* test. **C** Western blot showing the phosphorylation of AMPK protein in AICAR or DMSO treated KGN cells. For gray value quantification of proteins, data were normalized to the internal reference. Shown are mean ± SD. n.s. no significance, *p < 0.05 by two-tailed Student’s *t* test. **D** The CCK8 assay showed the cell viability of *ZNF674-AS1* silenced, AICAR treated and negative control KGN cells. Results are expressed as the mean ± SD (*n* = 3). **p < 0.01, ***p < 0.001 by two-tailed Student’s *t* test. **E**, **F** EdU staining assay showing the proliferation ability of *ZNF674-AS1* silenced, AICAR treated, and negative control KGN cells. Results are expressed as the mean ± SD (*n* = 3). **p < 0.01, ***p < 0.001 by two-tailed Student’s *t* test. Scale bar: 100 μm.

AMPK is a sensor monitoring availability of nutrients and energy and regulates cell growth^22^. Therefore, we investigated whether *ZNF674-AS1* regulates the activation of AMPK. Interestingly, the phosphorylation levels of AMPK Thr172 and its substrates ACC were elevated after *ZNF674-AS1* silencing (Fig. 5A, B). Furthermore, activation of AMPK by its activator Acadesine (AICAR) repressed the proliferation of KGN cells (Fig. 5C–F), which mimics the impact after *ZNF674-AS1* knockdown. These results suggested that *ZNF674-AS1* may execute its function by activating AMPK.

### *ZNF674-AS1* regulates the proliferation of GCs through ALDOA/v-ATPase-dependent AMPK activation

**Fig. 6:**
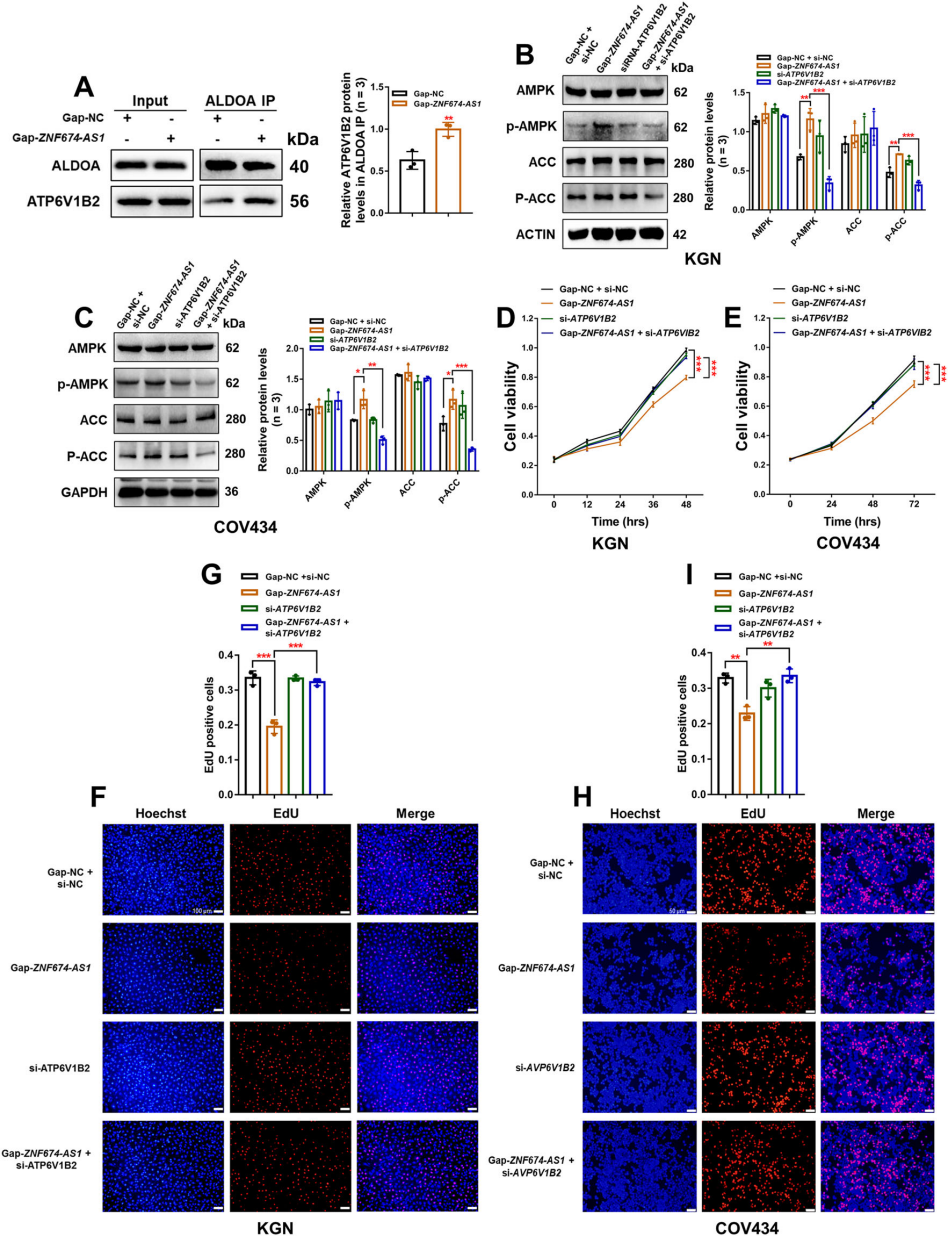
*ZNF674-AS1* regulates the proliferation of GCs through ALDOA/v-ATPase-dependent AMPK activation. **A** Western blot showing the protein level of ATP6V1B2 immunoprecipitated by anti-ALDOA antibodies in *ZNF674-AS1* silenced and negative control KGN cells. For gray value quantification of ATP6V1B2, data were normalized to ALDOA. Shown are mean ± SD. **p < 0.01 by two-tailed Student’s *t* test. **B**, **C** Western blot showing the levels of phosphorylated proteins involved in the AMPK signaling pathway in *ZNF674-AS1* silenced, *ATP6V1B2* silenced, co-silenced and negative control KGN and COV434 cells. For gray value quantification of proteins, data were normalized to the internal reference. Shown are mean ± SD. *p < 0.05, **p < 0.01, ***p < 0.001 by two-sided Student’s *t* test. **D**, **E** The CCK8 assay showed the cell viability of *ZNF674-AS1* silencing, *ATP6V1B2* silenced, co-silenced and negative control KGN and COV434 cells. Results are expressed as the mean ± SD (*n* = 3). ***p < 0.001 by two-tailed Student’s *t* test. **F**–**I** EdU staining assay showing the proliferation ability of *ZNF674-AS1* silenced, *ATP6V1B2* silenced, co-silenced and negative control KGN and COV434 cells. Results are expressed as the mean ± SD (*n* = 3). **p < 0.01, ***p < 0.001 by two-tailed Student’s *t* test. Scale bars: 100 μm (**F**) and 50 μm (**H**).

AMPK activation is a hierarchical process including AMP-dependent and AMP-independent mechanisms^23^. In AMP-independent mechanisms, ALDOA binding to v-ATPase is the major premise for the activation of AMPK that located at the lysosome^18,20^. Therefore, we explored whether *ZNF674-AS1* activates AMPK in an ALDOA/v-ATPase-dependent manner. We found that silence of *ZNF674-AS1* promoted ALDOA binds to ATP6V1B2 (Fig. 6A), a non-catalytic subunit of v-ATPase. Furthermore, knockdown of *ATP6V1B2* via siRNAs had no obvious impact on AMPK activation, however, abolished the activation effect of *ZNF674-AS1* silence on AMPK of KGN and COV434 cells (Supplementary Fig. 1G and Fig. 6B, C). These results revealed that *ZNF674-AS1* activates AMPK through the ALDOA/v-ATPase-dependent pathway. In addition, CCK8 and EdU assays showed that the inhibitory effect of *ZNF674-AS1* silence on KGN and COV434 proliferation was reversed by co-transfected *ATP6V1B2* siRNAs (Fig. 6D–I). Collectively, these data indicated that ALDOA/v-ATPase-dependent AMPK activation is responsible for the regulation of *ZNF674-AS1* on the proliferation of GCs.

## Discussion

**Fig. 7:**
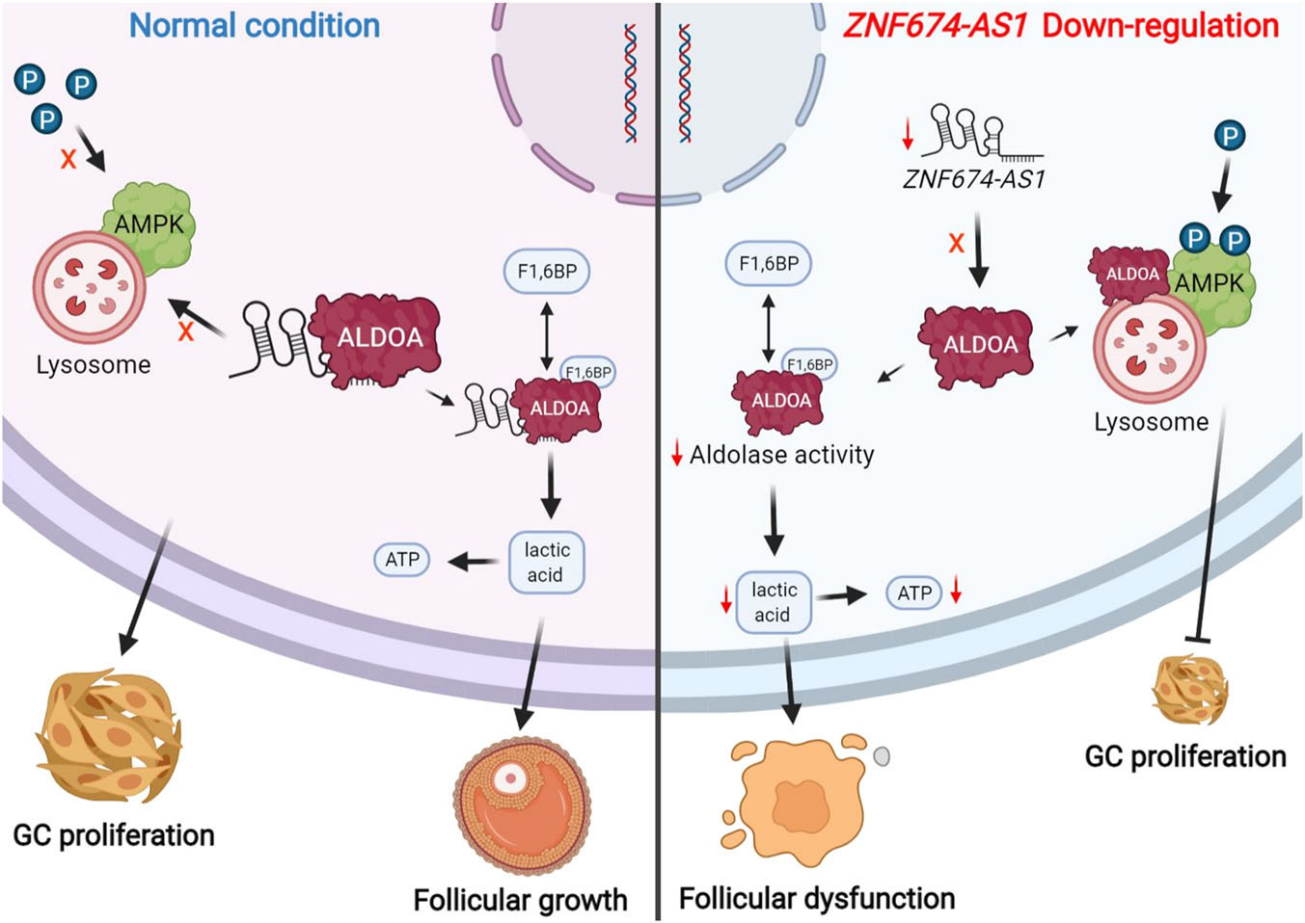
Graphical abstract describes that ZNF674-AS1 regulates the glycolysis and proliferation of GCs. Down-regulated *ZNF674-AS1* lead to the decreased enzymatic activity of ALDOA and promoted the activation of lysosome localized AMPK, therefore inhibiting the glycolysis and proliferation of GCs.

In this study, we reported a down-regulated lncRNA *ZNF674-AS1* in GCs from patients with bPOI, which was essential for the glycolysis and proliferation of GCs (Fig. 7). *ZNF674-AS1* was an energy stress-responsive lncRNA that functioned through binding with ALDOA in GCs. Decreased expression of *ZNF674-AS1* resulted in reduced enzymatic activity of ALDOA and promoted the activation of AMPK as well, therefore inhibiting the proliferation of GCs.

By convention, POI derives from the insufficient primordial follicle and accelerated follicle loss. Within follicles, GCs are the somatic cells to support and maintain the development of oocyte and follicle^2,3^. At the initiation of oocyte growth, flattened pregranulosa cells enclosed the oocytes to protect them from cell death^24^. Thereafter, GCs proliferate mitotically and undergo shape change so that they could provide sufficient materials, such as metabolic substrate, estradiol, to drive folliculogenesis and prevents follicular atresia^3^. In the present study, we showed that down-regulated lncRNA *ZNF674-AS1* significantly inhibited the proliferation of GCs. Similarly, lncRNA *GCAT1* has been shown to regulate GCs proliferation in patients with bPOI^25^. Our study provided new regulatory evidence conducted by lncRNA that mediates GCs proliferation.

We identified a novel interaction between lncRNA *ZNF674-AS1* and ALDOA, and showed that this lncRNA–protein complex was essential for the aldolase activity and glycolysis process in GCs. Glucose is the major substrate for ATP synthesis in GCs^26^. Of note, in order to minimize oxidative damage, glycolysis is the predominant energy metabolism pathway in oogonia and primordial follicle^27^. With regard to the course of oocyte maturation, oocyte up-regulates the expression of glycolytic genes in GCs to promote the production of pyruvate and lactate, which are readily utilized by the oocyte^28,29^. In the present study, *ZNF674-AS1* was inducible under energy stress and insufficient synthesis of lactate and ATP was observed in *ZNF674-AS1* silenced KGN cells. Therefore, follicular growth might be restrained in a state of low expression of *ZNF674-AS1*.

ALDOA is a central enzyme in the glycolysis process which catalyzes F1,6BP to glyceraldehyde 3-phosphate and dihydroxyacetone phosphate^17^. Several lncRNAs have been reported to mediate the expression of certain glycolytic enzymes^30,31^, suggesting the epigenetic modulation of glycolysis. However, we revealed a distinct mechanism that *ZNF674-AS1* regulates glycolysis via direct binding to ALDOA and modulating its aldolase activity without affecting its expression and subcellular distribution, which provided a better understanding of the mechanisms by which lncRNAs conduct their regulatory functions. Similarly, lncRNA *HULC* promotes glycolysis by modulating enzymatic activities of glycolytic enzymes in hepatocellular carcinoma cell lines^15^. To our knowledge, *ZNF674-AS1* is the first lncRNA involved in the aldolase activity of ALDOA that is involved in the metabolism homeostasis of GCs.

We demonstrated that aberrant AMPK activation is the downstream pathway of *ZNF674-AS1* regulating GCs proliferation. AMPK is a sensor of cellular energy status and a regulator of cell growth^23,32^. It is well established that AMPK inhibits the proliferation of different cell types including GCs^33,34^. Our present data re-emphasized the inhibitory impact of AMPK on cell growth. AMPK activation depends on its subcellular localization and levels of cellular AMP^19,20^. Under the early stage of energy deficiency, AMPK localized at lysosome is exclusively activated through the association of ALDOA with v-ATPase^20^. Our results showed that down-regulated *ZNF674-AS1* promoted ALDOA binding to v-ATPase, thereby activating AMPK. Furthermore, silencing ATP6V1B2, the non-catalytic subunit of v-ATPase, reversed the *ZNF674-AS1* silencing-induced activation of AMPK and inhibition of GCs proliferation. Collectively, our findings suggest that *ZNF674-AS1* exerts its function at least in part through regulating AMPK activation at lysosome.

In conclusion, we identified a down-regulated lncRNA *ZNF674-AS1* in GCs from patients with POI and demonstrated its regulatory function in the proliferation, glycolysis, and AMPK activation of GCs through interaction with ALDOA. Our study revealed a novel lncRNA–aldolase complex through which the lncRNA exerts its functions, providing new evidence of epigenetic regulation of GCs function and the development of POI.

## Materials and methods

### Clinical samples

33 women with bPOI and 41 age and BMI-matched control women undergoing in-vitro fertilization or intracytoplasmic sperm injection and embryo transfer treatment due to tubal obstruction or male factors in the Reproductive Hospital affiliated to Shandong University were selected in this study. bPOI is defined as follows: (i) <40 years of age; (ii) 10 IU/l < basal serum FSH < 25 IU/l; (iii) AMH < 1.1 ng/ml; and (iv) regular menstruation (every 23–35 days). Patients with chromosomal abnormalities or with a history of ovarian surgery, chemotherapy, or radiotherapy were excluded. The clinical characteristics of all participants are shown in Table 1.

**Table 1:** Clinical characteristics of participants.

Variables	Control (*n* = 41)	bPOI (*n* = 33)	*p* value
*Baseline characteristics*			
Age (year)	31.32 ± 3.88	32.67 ± 4.08	0.150^a^
BMI (kg/m^2^)	21.41 ± 1.92	21.45 (19.50, 24.16)	0.493^b^
Basal FSH (IU/l)	6.39 ± 1.19	12.63 (11.72, 17.10)	<0.0001^b^
Basal LH (IU/l)	5.53 ± 1.62	5.45 (4.29, 7.53)	0.652^b^
Basal E2 (pg/ml)	34.70 (24.35, 50.40)	35.93 ± 18.09	0.609^b^
AMH (ng/ml)	3.03 (2.42, 5.01)	0.59 ± 0.31	<0.0001^b^

Data are presented as mean ± SD or median (inter-quartile range (IQR) based on distribution.

^a^Student’s *t* test.

^b^Mann–Whitney *U*-test.

### RNA isolation and qRT-PCR analysis

GCs were collected and purified as previously described^35^. Total RNAs were isolated from GCs or KGN and COV434 cells using TRIzol reagent (Invitrogen, USA). The PrimeScript™ RT reagent Kit (TaKaRa, China) was applied to synthesize complementary DNA according to the manufacturer’s protocol. Quantitative RT-PCR was performed using SYBR Green Master Mix (TaKaRa), and *GAPDH* was used as a reference gene. Primer sequences are listed in Supplementary Table 2.

### Cell culture

The human GC-like tumor cell line KGN was obtained from the RIKEN BioResource Center (Japan)^36^. The human granulosa-like tumor cell line COV434 was obtained from Prof. Ying Xu of Nanjing University in China. Cells were cultured in DMEM (HyClone, USA) or DMEM/high glucose (COV434) supplemented with 10% FBS (Biological Industries, ISR) and 1% penicillin–streptomycin (Invitrogen). Cells were cultured at 37 °C in a humidified atmosphere containing 5% CO_2_.

### Cell transfection

Antisense LNA GapmeRs were obtained from QIAGEN. The siRNA duplexes were synthesized and purchased from GenePharma (China). KGN and COV434 cells were transfected with Antisense LNA GapmeRs and/or siRNAs using Lipofectamine 3000 Reagent (Invitrogen) according to the manufacturer’s protocols. The sequences of siRNA and Antisense LNA GapmeRs are provided in Supplementary Table 3.

### Cell viability assay

The viability of KGN and COV434 cells was evaluated using Cell Counting Kit-8 (CCK8, Beyotime, China) assays. 4000 cells were seeded and transfected in 96-well plates. Then, CCK-8 solution was added at the different time periods and the cells were incubated for 2 h at 37 °C. Finally, the absorbance was measured at 450 nm.

### EdU proliferation assay

For the cell proliferation assay, KGN or COV434 cells were seeded and transfected into 96-well plates. 48 h after transfection, the proliferation was detected through the EdU Cell Proliferation Assay Kit (EdU, Ribobio, China) according to the manufacturer’s instructions.

### Western blot

Cells were lysed in SDS buffer supplemented with proteinase inhibitor cocktail (Cell Signaling Technology, USA). The protein concentrations were determined with a BCA assay kit (Invitrogen). Approximately 20 μg cellular proteins were resolved by SDS–PAGE and transferred to polyvinylidene fluoride membranes (Millipore, USA). Specific primary antibodies were incubated with the membranes at 4 °C overnight followed by secondary antibodies (Proteintech, China). Then, membranes were incubated with the ECL chemiluminescence kit (Millipore) and imaged using ChemiDoc MP Imaging System (BIO-RAD, USA). The details of the antibodies are given in Supplementary Table 4.

### Cytoplasm/nucleus fractionation

Cytoplasmic and nuclear fractions were prepared using a PARIS^TM^ Kit (Invitrogen) according to the manufacturer’s instructions, and the RNAs were then subjected to qRT-PCR analysis.

### Biotin-labeled RNA pulldown assay

RNA was transcribed in vitro using the MEGAscrip T7 Transcription Kit (Invitrogen) and biotinylated using the Pierce RNA 3′ End Desthiobiotinylation Kit (Invitrogen) according to the manufacturers’ instructions. Then, RNA pull-down assays were performed using the Pierce Magnetic RNA–Protein PullDown Kit according to the manufacturer’s instructions. Co-precipitated proteins were subjected to MS analysis or western blot.

### RNA immunoprecipitation (RIP)

RIP assay was performed using the EZ-Magna RIP kit (Millipore) according to the manufacturer’s instructions. Cellular proteins from KGN cells were lysed in RIP lysis buffer with RNase inhibitor and protease inhibitor cocktail. Then, cell lysates were incubated with specific primary antibodies at 4 °C overnight. Simultaneously, the same amount of cellular proteins were incubated with A homologous IgG. The co-precipitated RNA was extracted using TRIzol reagent and quantified by qRT-PCR as described above. The details of the antibodies are given in Supplementary Table 4.

### Immunofluorescence staining

KGN cells were fixed in 4% paraformaldehyde at room temperature for 20 min. Cells were then permeabilized and blocked in 0.3% Triton X-100/10% BSA/PBS for 30 min. Incubation for primary antibody was done at 4 °C overnight followed by incubation with Alexa Fluor secondary antibody for 1 h at room temperature in the dark. The details of the antibodies are given in Supplementary Table 4.

### Measurements of aldolase activity, F1, 6BP, lactate, ATP, and ADP levels

The aldolase activity was determined by Aldolase Activity Colorimetric Assay Kit (BioVision, USA) according to the manufacturer’s protocol. The Lactate Colorimetric Assay Kit II (BioVision) and the PicoProbeTM Fructose-1,6-Bisphosphate Assay Kit (BioVision) were applied to detect the levels of lactate and F1,6BP. The intercellular ATP levels were detected by ATP detection kit (Beyotime) and ADP/ATP ratio was determined using ApoSENSOR™ ADP/ATP Ratio Bioluminescent Assay Kit (BioVision) according to manufacturer’s instructions.

### AMPK activation assay

AICAR was used as a pharmacological activator of AMPK, which was purchased from APExBIO (USA). KGN cells were treated with AICAR at the final concentration of 0.1 mM for 24 h to evaluate the effect of AMPK activation.

### Co-immunoprecipitation assay

KGN cells were lysed in Pierce IP lysis buffer. The lysates were incubated overnight at 4 °C with specific primary antibodies followed by binding to PureProteom Protein A/G Magnetic Beads (Millipore) at room temperature for 1 h. The immunoprecipitates were then washed and applied to western blot. The details of the antibodies are given in Supplementary Table 4.

### Statistics

Statistical analysis was performed using the SPSS software and GraphPad Prism 8 (GraphPad Software, USA). The statistical significance was analyzed by the two-tailed Student’s *t* test, and the Mann–Whitney *U* test. Pearson correlation and linear regression analyses were used to determine the correlation between *ZNF674-AS1* and clinical characteristics in the bPOI group (*n* = 33) or all participants (bPOIs and Controls, *n* = 74).

## Supplementary information

supplementary Figure Legends

Supplementary figure 1

supplementary Table 1 Differentially expressed lncRNAs in GCs from patients with bPOI

Supplementary Table 2 List of primers used in this study

Supplementary Table 3 List of LNA GapmeRs and siRNA used in this study

Supplementary Table 4 List of antibodies used in this study
