# Supplementary material for: LncRNA ZNF674-AS1 regulates granulosa cell glycolysis and proliferation by interacting with ALDOA
Source: Cell Death Discov. 2021 May 16;7:107. doi: 10.1038/s41420-021-00493-1 (PMC8124069; doi:10.1038/s41420-021-00493-1)
Supplement: Supplementary file 1 — supplementary Figure Legends [file 41420_2021_493_MOESM1_ESM.docx]

**Supplementary Fig. 1.** (A) The correlation between the expression level of *ZNF674-AS1* in GCs and the serum concentration of AMH, FSH from patients with bPOI (n = 33) was analyzed by Pearson correlation analysis. (B) The coding capacity of *ZNF674-AS1* was assessed by Coding-Potential Assessment Tool (CPAT). (C) The efficiency of *ZNF674-AS1* silencing in KGN and COV434 cells was measured by qRT-PCR. Results are expressed as the mean ± SD (n = 3). ***p < 0.001 by two-tailed Student’s t-test. (D) The estradiol (E2) level was measured in *ZNF674-AS1*-silenced and negative control KGN cells. Results are expressed as the mean ± SD (n = 3). n.s. = no significance by two-tailed Student’s t-test. (E) Western blot showing the protein levels of FSHR and CYP19A1 in *ZNF674-AS1* silenced and negative control KGN cells. For gray value quantification of proteins, data were normalized to the internal reference. Shown are mean ± SD. n.s. = no significance by two-tailed Student’s t-test. (F) Immunofluorescence assay showing the subcellular localization of ALODA in *ZNF674-AS1* silenced and negative control KGN cells. Scale bar: 10 μm. (G) The efficiency of *ATP6V1B2* silencing in KGN and COV434 cells was measured by western blot. For gray value quantification of ATP6V1B2, data were normalized to the internal reference. Shown are mean ± SD (n = 3). *p < 0.05, ***p < 0.001 by two-tailed Student’s t-test.
