# Supplementary figures and images for: LncRNA ZNF674-AS1 regulates granulosa cell glycolysis and proliferation by interacting with ALDOA
Source: Cell Death Discov. 2021 May 16;7:107. doi: 10.1038/s41420-021-00493-1 (PMC8124069; doi:10.1038/s41420-021-00493-1)

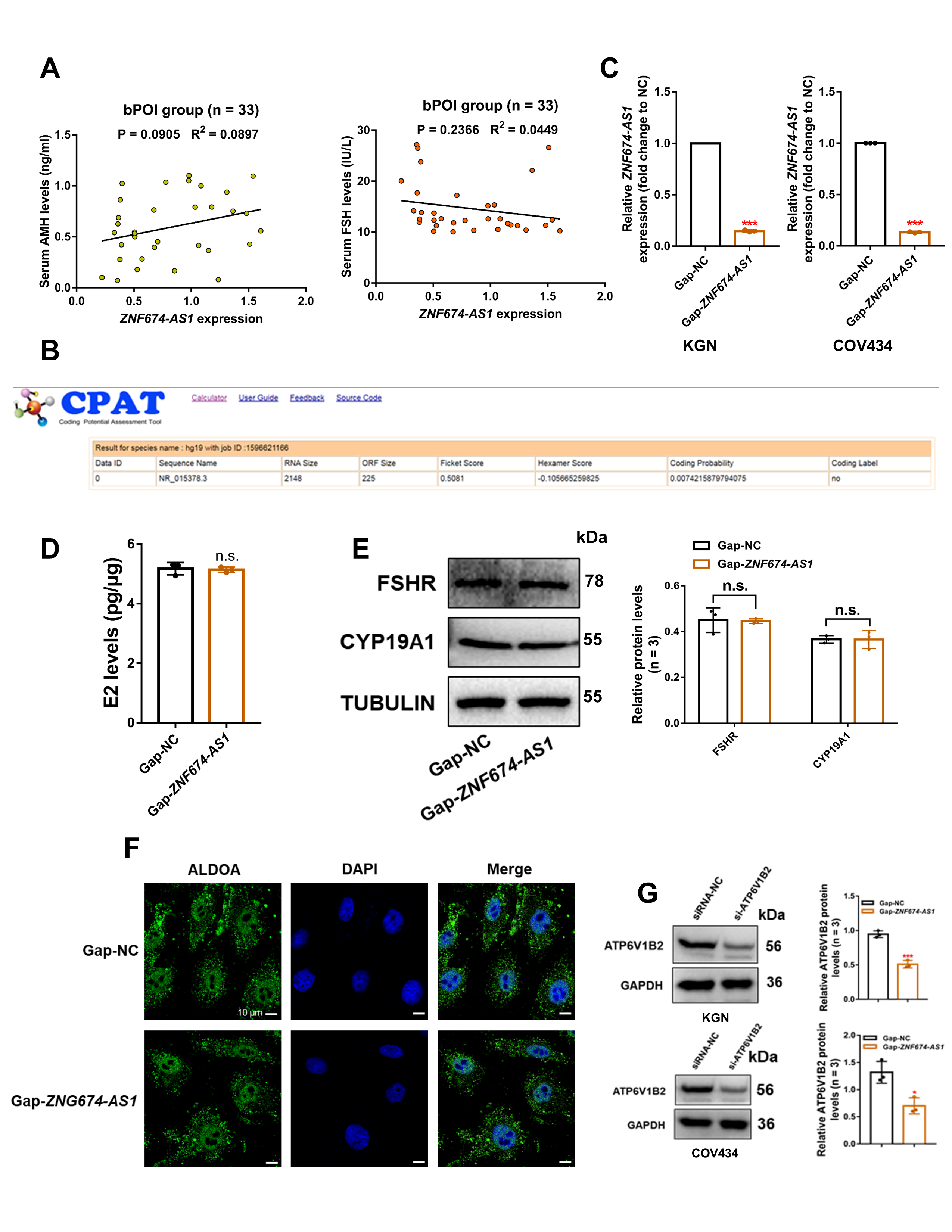

Supplement: Supplementary file 2 — Supplementary figure 1 [file 41420_2021_493_MOESM2_ESM.tif]
