# Supplementary material for: LncRNA ZNF674-AS1 regulates granulosa cell glycolysis and proliferation by interacting with ALDOA
Source: Cell Death Discov. 2021 May 16;7:107. doi: 10.1038/s41420-021-00493-1 (PMC8124069; doi:10.1038/s41420-021-00493-1)
Supplement: Supplementary file 4 — Supplementary Table 2 List of primers used in this study [file 41420_2021_493_MOESM4_ESM.docx]

**Supplementary Table 2 List of primers used in this study**

| **Gene**  **(homo sapiens)** | **Forward (5'-3')** | **Reverse (5'-3')** |
| --- | --- | --- |
| *ZNF674-AS1* | CCATGCCAGCATTTGCTATTCA | GGGCTTGAGGCTCTAAAGATGT |
| *GAPDH* | GGGAAACTGTGGCGTGAT | GAGTGGGTGTCGCTGTTGA |
| *LMNB1* | GAAAAAGACAACTCTCGTCGCA | GTAAGCACTGATTTCCATGTCCA |
| *MALAT1* | AAAGTCCGCCATTTTGCCAC | CTCACAAAACCCCCGGAACT |
| *β-actin* | ACAGAGCCTCGCCTTTGCC | GAGGATGCCTCTCTTGCTCTG |
