# Supplementary material for: LncRNA ZNF674-AS1 regulates granulosa cell glycolysis and proliferation by interacting with ALDOA
Source: Cell Death Discov. 2021 May 16;7:107. doi: 10.1038/s41420-021-00493-1 (PMC8124069; doi:10.1038/s41420-021-00493-1)
Supplement: Supplementary file 5 — Supplementary Table 3 List of LNA GapmeRs and siRNA used in this study [file 41420_2021_493_MOESM5_ESM.docx]

**Supplementary Table 3 List of LNA GapmeRs and siRNA used in this study**

| **siRNAs** | **Sense(5'-3')** |
| --- | --- |
| Gap-*ZNF674-AS1* | CTGAAGAGATTGGCGC |
| siRNA-*ATP6V1B2* | GCUACCACACAAUGAGAUUTT |
