# Supplementary material for: LncRNA ZNF674-AS1 regulates granulosa cell glycolysis and proliferation by interacting with ALDOA
Source: Cell Death Discov. 2021 May 16;7:107. doi: 10.1038/s41420-021-00493-1 (PMC8124069; doi:10.1038/s41420-021-00493-1)
Supplement: Supplementary file 6 — Supplementary Table 4 List of antibodies used in this study [file 41420_2021_493_MOESM6_ESM.docx]

**Supplementary Table 4 List of antibodies used in this study**

| **Antibody** | **Supplier** | **Catalog#** | **Application** |
| --- | --- | --- | --- |
| Anti-PCNA | Proteintech | 10205-2-AP | WB |
| Tubulin | Proteintech | 66031-1-Ig | WB |
| Anti-ALDOA | Cell Signaling Technology | #8060 | WB |
| Anti-ALDOA | SANTA CRUZ | sc-390733 | IF, RIP, IP |
| Actin | Proteintech | 66009-1-Ig | WB |
| Anti-AMPK | Cell Signaling Technology | #9957 | WB |
| Anti-p-AMPK | Cell Signaling Technology | #9957 | WB |
| Anti-ACC | Cell Signaling Technology | #9957 | WB |
| Anti-p-ACC | Cell Signaling Technology | #9957 | WB |
| GAPDH | Proteintech | 60004-1-Ig | WB |
| Anti-ATP6V1B2 | Abcam | ab73404 | WB |
| Anti-FSHR | Proteintech | 22665-1-AP | WB |
| Anti-CYP19A1 | Cell Signaling Technology | #14528 | WB |
